# Circulating Adiponectin Is Associated with Renal Function Independent of Age and Serum Lipids in West Africans

**DOI:** 10.1155/2012/730920

**Published:** 2012-08-22

**Authors:** A. P. Doumatey, J. Zhou, H. Huang, J. Adeleye, W. Balogun, O. Fasanmade, T. Johnson, J. Oli, G. Okafor, A. Amoah, B. Eghan, K. Agyenim-Boateng, J. Acheampong, C. Adebamowo, A. Adeyemo, C. N. Rotimi

**Affiliations:** ^1^Center for Research on Genomics and Global Health, National Human Genome Research Institute, National Institutes of Health, Bethesda, MD 20852, USA; ^2^Department of Medicine, University of Ibadan, Ibadan 200284, Nigeria; ^3^Department of Medicine, University of Lagos, Lagos 101017, Nigeria; ^4^Department of Medicine, University of Nigeria Teaching Hospital, Enugu 400001, Nigeria; ^5^Department of Medicine and Therapeutics, University of Ghana Medical School, Accra, Ghana; ^6^Department of Medicine, University of Science and Technology, Kumasi, Ghana; ^7^Institute of Human Virology, School of Medicine, University of Maryland, Baltimore, MD 21201, USA

## Abstract

Adiponectin, a protein secreted by adipose tissue, has been associated with renal dysfunction. However, these observations have not been adequately investigated in large epidemiological studies of healthy individuals in general and in African populations in particular. Hence, we designed this study to evaluate the relationship between adiponectin and renal function in a large group of nondiabetic West Africans. Total adiponectin was measured in 792 participants. MDRD and Cockroft-Gault (CG-) estimated GFR were used as indices of renal function. Linear and logistic regression models were used to determine the relationship between adiponectin and renal function. Adiponectin showed an inverse relationship with eGFR in univariate (Beta_MDRD_ = −0.18, Beta_CG_ = −0.26) and multivariate (Beta_MDRD_ = −0.10, Beta_CG_ = −0.09) regression analyses. The multivariate models that included age, sex, BMI, hypertension, smoking, HDL-C, LDL-C, triglycerides, and adiponectin explained 30% and 55.6% of the variance in GFR estimated by MDRD and CG methods, respectively. Adiponectin was also a strong predictor of moderate chronic kidney disease (defined as eGFR < 60 mL/min/1.73 m^2^). We demonstrate that adiponectin is associated with renal function in nondiabetic West Africans. The observed relationship is independent of age and serum lipids. Our findings suggest that adiponectin may have clinical utility as a biomarker of renal function.

## 1. Introduction

Adiponectin, a 30 kDa protein secreted mainly by adipose tissue into the bloodstream, has been associated with a number of diseases [1], including metabolic disorders such as obesity and type 2 diabetes (T2D) [2–4]. More recently, the relationship between adiponectin and kidney function is gaining increasing recognition [5–8], although most of the evidence linking adiponectin to kidney function has come from studies of individuals with disease [6, 7, 9–11]. Serum adiponectin is elevated in persons with impaired kidney function but this becomes normal following kidney transplantation [12]. However, the relationship between adiponectin and kidney function is complex. For example, low adiponectin level is associated with T2D at the onset but adiponectin levels seem to increase with increasing duration of T2D and in diabetic patients with nephropathy [6]. These observations suggest that circulating levels of adiponectin are heavily influenced by the pathophysiologic state of individuals as a result of multiple mechanisms, including reduced clearance by the kidney [13] and degree of sensitivity or resistance to adiponectin [14, 15].

Adiponectin has been suggested as a potential kidney biomarker in chronic kidney disease (CKD) progression [12]. However, the studies linking adiponectin and renal function have two potential limitations. First, the results and conclusions of these studies have been inconsistent and contradictory [8, 16–18]. Second, most of the studies investigating the relationship between adiponectin and kidney function have been conducted in persons with existing metabolic disorders (e.g., diabetes). To our knowledge, there are only two studies [16, 19] of healthy individuals (i.e., asymptomatic individuals, not previously diagnosed with any serious or chronic illnesses with the exception of hypertension). Given the increasing importance of kidney diseases in sub-Saharan Africa and the young age (20–50 years) at which CKD is often seen [20], investigating the potential utility of adiponectin as a biomarker for renal function in these populations is warranted.

We have previously investigated the relationship between adiponectin levels, obesity, and metabolic markers in West Africans and showed that adiponectin was significantly associated with age, BMI, waist circumference, and serum lipids [21]. Since the relationship between adiponectin and renal function seems to be confounded by disease status, we reasoned that it would be informative to study this relationship in a cohort without any known major morbidity. In the present study, we aimed to investigate the relationship between renal function as measured by estimated glomerular filtration rate (eGFR) and adiponectin in nondiabetic West Africans.

## 2. Subjects and Methods

### 2.1. Study Population

This study included nondiabetic West Africans aged 18–79 years who participated in the Africa America Diabetes Mellitus (AADM) study. The AADM study, a T2D project ongoing in West Africa, is designed to investigate the genetic and environmental determinants of T2D and associated comorbidities, including obesity, kidney function, and several biochemical markers of metabolic disorders [22]. Briefly, the AADM study was conducted in three centers in Nigeria (Enugu, Ibadan, and Lagos) and two centers in Ghana (Accra and Kumasi) as part of an international collaboration between US investigators and West African Scientists. The AADM study enrolled both families and unrelated persons with and without diabetes (controls). The study protocol was approved by the IRB of each participating West African institution, and participants gave their informed consents following the Helsinki guidelines. Participants with an eGFR ≤ 30 mL/min⁡ per 1.73 m^2^ were excluded from this study. The final study sample consisted of 792 qualified participants.

### 2.2. Anthropometric and Clinical Measurements

The study used standardized protocols for anthropometric (height, weight, and waist circumference) and clinical measurements. Weight was measured in light clothing using an electronic scale to the nearest 0.1 kg, height was obtained using a stadiometer to the nearest 0.1 cm, and waist circumference was measured to the nearest 0.1 cm at the narrowest part of the torso. Blood pressure was measured in the sitting position using an oscillometric device (Omron). Fasting serum samples were used to measure number of clinical biomarkers including creatinine, HDL-C, LDL-C, and triglycerides using an autoanalyzer, COBAS Integra 400 plus (Roche Diagnostics, Indianapolis, IN); creatinine was measured using a modified Jaffé reaction. Total serum adiponectin was measured using a commercially available immunoassay kit (R&D Systems, Minneapolis, MN) following the manufacturer's instructions. eGFR was used as measure of kidney function. The estimation of GFR remains controversial especially in Africans [23] because available methods have not been validated in African or Asian populations. In the absence of generalizable methods in African populations, we choose to estimate GFR in this study by using two distinct calculation methods: (1) the Modification of Diet in Renal Diseases (MDRD) formula, that is widely used by clinicians and researchers [24] as follows: eGFR_MDRD_ = 186 × creatinine^(−1.154)^ × age^(−0.203)^ × 0.742 (if female) × 1.21 (if Blacks), and (2) Crockroft-Gault (CG) calculation as follows [25]: eGFR_CG_ = (140 − age) × body  weight/plasma  creatinine ×72 (×0.85 if female). To compare GFR estimated by MDRD and CG methods, the CG formula was normalized per 1.73 m^2^ of body surface area (BSA) estimated by the formula of Du Bois and Du Bois [26], BSA = (body  weight^0.425^  (in  kg) × height^0.725^  (in  cm)) × 0.007184. Using the guidelines of the National Kidney Foundation Kidney Disease Outcome Quality Initiative [27], we defined moderate CKD as eGFR < 60 mL/min/1.73 m^2^. The reference group (i.e., participants with “normal” eGFR) was individuals with eGFR ≥ 60 mL/min per 1.73 m^2^.

### 2.3. Statistical Analysis

All statistical analyses were carried out using *SPSS* package, version 16.0. Normality of all variables was checked using Q-Q plots. Variables with skewed distributions (adiponectin, creatinine, HDL-C, LDL-C, TG, eGFR_MDRD_, and eGFR_CG_) were logarithmically transformed to prevent violation of normality assumptions. Continuous variables were expressed as mean ± standard deviation unless otherwise specified; Student's-*t*  test was used to compare mean between groups. Partial Pearson's correlation coefficients and simple and multiple linear regression models were used to evaluate the association between renal function (eGFR_MDRD_ and eGFR_CG_) and adiponectin. In these models, eGFR_MDRD_ or eGFR_CG_ was the dependent variable, and adiponectin was the explanatory variable. Adjustment was made for factors that are known to affect kidney function specifically age, sex, BMI, hypertension, smoking, and serum lipids (triglycerides (TG), HDL-cholesterol (HDL-C), and LDL-cholesterol (LDL-C)). Hypertension, smoking, and sex were included in the models as categorical variables. Using logistic regression models, we determined the predictors of eGFR_MDRD_ and eGFR_CG_ in moderate CKD (i.e., eGFR < 60 mL/min per 1.73 m^2^). The first logistic model was an unadjusted model in which only adiponectin levels were the predictor; in the second model, we adjusted for age and sex, and, finally, the third logistic model included the lipid parameters (HDL-C, LDL-C, and TG). Significance level was set at 0.05 for all analyses.

## 3. Results

The 792 subjects comprised 315 men (mean age 48.3 ± 14.2 years) and 477 women (mean age 45.1 ± 12.0 years). The characteristics of this cohort are presented in Table 1. Overall, men had lower BMI, waist circumference, and adiponectin levels (Table 1(a)). The two methods used to estimate GFR in this study (MDRD and CG) are highly correlated (*r*_*p*_ = 0.90,  *P* < 0.0001). Despite this high degree of correlation, when the MDRD method is used to estimate GFR, the prevalence of moderate CKD is 8.5%, a value that almost doubles when the CG method is used (16.3%). The characteristics of the study participants categorized by eGFR are summarized in Table 1(b). On average, GFR estimated by the MDRD method was higher compared to that by CG method (100.4 versus 90.4 min/mL per 1.73 m^2^). Participants with moderate CKD (i.e., eGFR < 60 mL/min per 1.73 m^2^) were significantly older and had an elevated serum creatinine as well as serum adiponectin (based on MDRD: 8511.4 ng/mL versus 6760.8 ng/mL and based on CG: 8709.6 ng/mL versus 6606.9 ng/mL).

**Figure 1:**
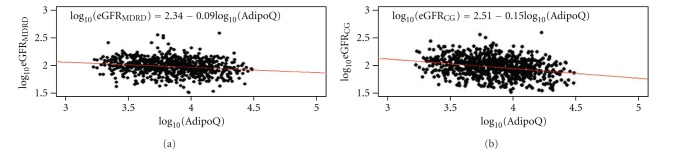
Scatter plots of log transformed eGFR (vertical axis) versus log adiponectin (horizontal axis) in nondiabetic West Africans. (a) Relationship between GFR estimated by MDRD method (eGFR_MDRD_) and adiponectin, (b) relationship between GFR estimated by Cockroft-Gault (CG) method (eGFR_CG_) and adiponectin. A linear regression line is superimposed on each plot to show the relationship with eGFR.

To explore the relationships between eGFR, adiponectin, and potential confounders, we first developed scatter plots (Figure 1) and calculated partial Pearson's coefficients of correlation (Table 2). The same variables were associated with both eGFR_MDRD_ and eGFR_CG_. eGFR was negatively associated with adiponectin (*P* < 0.01), age (*P* < 0.01), serum lipids (LDL-C, HDL-C, TG), and hypertension (*P* < 0.01). No association was found between eGFR and smoking (*P* = 0.68) in this cohort (Table 2). In contrast, adiponectin was positively associated with age, HDL-C, and hypertension (Table 2). Interestingly the association between eGFR_CG_ and adiponectin was stronger compared to the association between eGFR_MDRD_ and adiponectin (*r*_*p*_ = −0.35 versus *r*_*p*_ = −0.19).

In unadjusted linear regression model, adiponectin explained 3% of the variation in eGFR_MDRD_ (beta = −0.18,  *P* < 0.0001) whereas it explained about 7% of the variation in eGFR_CG_ (beta = −0.26,  *P* < 0.0001). In a multivariate model that included age, sex, BMI, hypertension, and serum lipids, adiponectin remained a significant predictor of eGFR_MDRD_ (beta = −0.10,  *P* = 0.004) and eGFR_CG_ (beta = −0.09, *P* = 0.001) as well as age (*P* < 0.0001) and serum lipids (*P* < 0.0001); this multivariate model explained 30% of the variation in eGFR_MDRD_ (Table 3). Additionally BMI and sex were also significantly associated with eGFR_CG_, and the variance explained why eGFR_CG_ increased to 55.6%. Furthermore, adiponectin was significantly associated with moderate CKD regardless of the estimation method used to define moderate CKD (*B*_MDRD_ = 1.61,  *P*_MDRD_ = 0.006 and *B*_CG_ = 1.90,  *P* < 0.0001, (Table 4)), and these associations are independent of the effects of serum lipids, age, and sex.

## 4. Discussion

We investigated the relationship between renal function and adiponectin in a large cohort of nondiabetic West Africans. Due to the fact that none of the GFR estimation equations has been formally validated in African populations we implemented the two most commonly used methods (MDRD and the Cockroft-Gault) in this study. The MDRD method is widely used in clinical practice, and the Cockroft-Gault method is the first creatinine-based method adopted by clinicians [28]. Despite the high correlation (*r*_*p*_ = 90%) between these two methods in our study, the CG-estimated GFR identified more individuals as having low eGFR (<60 mL/min per 1.73 m^2^) than the MDRD-estimated eGFR (16.3% versus 8.5%). Our finding corroborates with previous observations in other populations [29, 30]. For example, Wetmore et al. found that 5.3% of the subjects in their study were classified by the MDRD equation as having CKD (eGFR < 60 mL/min per 1.73 m^2^) whereas 19.7% were classified by the CG formula [29].

Regardless of the method used to estimate GFR, we observed a negative association between eGFR and circulating adiponectin, as has been previously reported by other investigators [8, 13, 31, 32]. However, most previous observations have been among persons with existing diseases such as type 1 and 2 diabetic, coronary disease and CKD. Thus, apart from confirming previous findings our study demonstrates that the inverse relationship between eGFR and adiponectin extends to persons with no obvious metabolic disorders. Additionally, we observed that the association between eGFR and adiponectin was stronger when Cockroft-Gault calculation was used as marker of renal function. While the reason for the observed difference is not obvious, we posit that it may be due to the fact that the CG equation takes into account anthropometric measurements—which also influence adiponectin levels—while the MDRD formula does not [28, 33].

Adiponectin levels alone explained 3% to 7% of the variation in eGFR in this cohort depending on the estimation method. Interestingly, in multiple regression analysis adjusting for additional factors such as smoking, sex, hypertension, and lipids, the variance explained increased by 8-fold to 56% for eGFR_CG_  and by 10-fold to 30% for eGFR_MDRD_. This dramatic increase in the percent variance explained was due primarily to the inclusion of age, sex, BMI, and serum lipids (HDL-C, LDL-C and triglycerides) in the regression models. The relationship between age, serum lipids and eGFR is well documented [34–37]. For example, baseline serum lipids (total cholesterol and LDL-C) are independent risk factors for renal diseases as well as powerful predictors of renal function [38]. It is therefore noteworthy that adiponectin remained a significant independent predictor of eGFR in the presence of age and serum lipids.

We also found adiponectin to be independently associated with moderate CKD (Table 4). These observations suggest that adiponectin may serve as a potential biomarker of kidney function even among individuals who do not have overt T2D. Furthermore, it provides additional evidence to support evaluating adiponectin along with other plasma proteins such as apolipoprotein A-IV, fibroblast growth factor 23, neutrophil gelatinase-associated lipocalin, and the natriuretic peptides as a discriminatory biomarker of CKD progression [12]. To fully understand the potential role of adiponectin in routine clinical pathology, more population-based studies as well as studies of adiponectin isomers in kidney function are needed. Kawamoto et al. [16] found a positive association between high molecular weight (HMW) adiponectin, and GFR, raising an important question about the role of adiponectin isomers in kidney function. HMW adiponectin has been shown to be the most potent form of adiponectin in its insulin sensitizing function [39, 40]. It is also likely that HMW adiponectin may offer more protection in the context of kidney function as suggested by the Japanese study of persons with mild CKD [16].

Despite our careful efforts to evaluate the relationship between adiponectin and renal function in this cohort, the design of the study (cross-sectional) and the use of eGFR as measure of renal function in healthy individuals are potential limitations of the study. A cross-sectional study does not permit the determination of causality in a relationship. Therefore, longitudinal studies are needed to provide such data. Secondly, MDRD-based estimates of eGFR may not be suitable for all populations, especially Africans, Asians [23], and individuals suffering from malnutrition or eating a vegetarian or low-meat diet. While our cohort did not include individuals with malnutrition, our study did not collect dietary data, and we could therefore not control for dietary factors in our analyses. Additionally, our definition of moderate CKD was solely based on eGFR, a creatinine-based estimation. No other kidney function markers such as proteinuria or urine albumin/creatinine were available in this study to explain the impaired kidney function seen in the subjects with low eGFR.

In summary, we demonstrated that adiponectin is an independent predictor of both eGFR and moderate CKD in nondiabetic West Africans. This confirms and extends previous findings of an inverse relationship between adiponectin and renal function. The uniqueness of our study lies in the inclusion of a healthier and younger West African population characterized by a lower prevalence of CKD. These attributes allowed us to evaluate the relationship between adiponectin and renal function across a larger age spectrum in a population that is generally understudied.

## Figures and Tables

**Table:** (a) Participant characteristics categorized by gender

Variable	All (*n* = 792)	Male (*n* = 315)	Female (*n* = 477)	*P* value
Age (years)	46.4 ± 13.0	48.3 ± 14.2	45.1 ± 12.0	0.0012
BMI (kg/m^2^)	25.8 ± 5.5	23.6 ± 3.99	27.3 ± 5.9	<0.0001
WC (cm)	87.8 ± 12.0	85.9 ± 10.86	89.1 ± 12.6	0.0002
Smoking (yes/no) (%)	4.8	8.3	2.5	0.0002
HTN (yes/no) (%)	35.1	35.9	34.6	0.71
CKD_MDRD_ (yes/no) (%)	8.5	9.2	8.0	0.54
CKD_CG_ (yes/no) (%)	16.3	23.8	11.1	<0.0001
Creatinine (mg/dL)				
Mean	1.0 ± 0.3	1.1 ± 0.3	0.9 ± 0.2	<0.0001
Geometric mean	0.9	1.1	0.8	
eGFR_MDRM_ (mL/min per 1.73 m^2^)				
Mean	100.4 ± 35.3	99.0 ± 34.5	101.3 ± 35.9	0.37
Geometric mean	95.5	93.3	95.5	
eGFR_CG_ (mL/min per 1.73 m^2^)				
Mean	90.4 ± 32.2	80.0 ± 28.0	97.2 ± 33.0	<0.0001
Geometric mean	85.1	75.9	91.2	
HDL-C (mg/dL)				
Mean	42.6 ± 18.5	41.4 ± 17.3	43.4 ± 19.2	0.15
Geometric mean	38.0	37.2	38.0	
LDL-C (mg/dL)				
Mean	130.3 ± 47.2	124.4 ± 48.2	134.1 ± 46.1	0.005
Geometric mean	123.0	114.8	125.9	
TG (mg/dL)				
Mean	91.1 ± 43.6	94.8 ± 50.3	88.7 ± 38.5	0.07
Geometric mean	83.2	87.1	81.3	
AdipoQ (ng/mL)				
Mean	8271.1 ± 5207.9	6956.0 ± 4525.8	9139.5 ± 5445.9	<0.0001
Geometric mean	6918.3	5888.4	7762.5	

BMI: body mass index; AdipoQ: adiponectin; eGFR: estimated glomerular filtration rate; WC: waist circumference; HTN: hypertension; CKD: chronic kidney disease defined as eGFR < 60 mL/min/1.73 m^2^; HDL-C: high density lipoproteins; LDL-C: low density lipoproteins; TG: triglycerides.

Results are expressed as mean ± standard deviation except when specified.

Student's *t*-test was used to compare means between groups and chi-square to compare frequencies between groups.

**Table:** (b) Characteristics of participants categorized by eGFR_MDRD_ and eGFR_CG_

Variable	eGFR_MDRD_ ≥ 60 *n* = 725	eGFR_MDRD_ < 60 *n* = 67	*P* value	eGFR_CG_ ≥ 60 *n* = 659	eGFR_CG_ < 60 *n* = 128	*P* value
Age (years)	45.8 ± 13.2	53.2 ± 9.2	<0.0001	44.1 ± 12.1	58.0 ± 11.2	<0.0001
BMI (kg/m^2^)	25.9 ± 5.6	25.5 ± 4.5	0.6	26.4 ± 5.7	22.6 ± 3.3	<0.0001
Waist circumference (cm)	87.6 ± 12.1	89.9 ± 10.3	0.13	88.4 ± 12.4	84.9 ± 9.3	<0.0001
Smoking (yes/no) (%)	5 (36/725)	3 (2/67)	0.47	5.3 (35/659)	2.3 (3/128)	0.16
HTN (yes/no) (%)	33.7	50.7	0.009	31.3	55.5	<0.0001
Male/female ratio	286/439	29/38	—	237/422	75/53	—
Creatinine (mg/dL)						
Mean	0.9 ± 0.2	1.6 ± 0.3	<0.0001	0.9 ± 0.2	1.4 ± 0.3	<0.0001
Geometric mean	0.9	1.6		0.9	1.3	
eGFR						
Mean	104.9 ± 33.52	52.3 ± 6.6	<0.0001	98.2 ± 29.2	50 ± 6.8	<0.0001
Geometric mean	100	51.3		95.5	49.0	
HDL-C (mg/dL)						
Mean	41.9 ± 18.0	49.7 ± 22.4	0.008	41.7 ± 17.7	47.6 ± 21.5	0.005
Geometric mean	37.2	43.7		36.3	42.7	
LDL-C (mg/dL)						
Mean	126.2 ± 43.5	174.4 ± 61.0	<0.0001	126.0 ± 43.9	152.8 ± 56.4	<0.0001
Geometric mean	117.5	162.2		117.5	141.3	
TG (mg/dL)						
Mean	88.26 ± 41.3	122.09 ± 54.8	<0.0001	88.7 ± 43.7	104.2 ± 41.6	<0.0001
Geometric mean	79.4	112.2		81.3	97.7	
AdipoQ						
Mean	8091.0 ± 5092.1	10220.2 ± 6032.9	0.007	7884.9 ± 4941.0	10376.2 ± 6054.6	<0.0001
Geometric mean	6760.8	8511.4		6606.9	8709.6	

BMI: body mass index; AdipoQ: adiponectin; eGFR: estimated glomerular filtration rate; WC: waist circumference; HTN: hypertension; CKD: moderate chronic kidney disease defined as eGFR < 60 mL/min/1.73 m^2^;HDL-C: high density lipoproteins; LDL-C: low density lipoproteins; TG: triglycerides.

Results are expressed as mean ± standard deviation except when specified.

Student's *t*-test was used to compare means between groups and chi-square to compare frequencies between groups.

**Table 2:** Pearson's coefficients of correlation (*r*_*p*_) between eGFR_MDRD_ or eGFR_CG_, adiponectin and other confounders adjusted for gender.

	log10AdipoQ	log10LDL	log10HDL	log10TG	log10GFR_MDRD_	log10GFR_CG_	HTN	BMI	SMOKING
log10LDL	0.08								
log10HDL	0.20	0.20							
log10TG	−0.08	0.40	−0.12						
log10GFR_MDRD_	−0.19	−0.38	−0.19	−0.37					
log10GFR_CG_	−0.35	−0.32	−0.18	−0.30	0.9				
HTN	0.15	0.19	0.14	0.21	−0.22	−0.28			
BMI	−0.30	0.17	NS	0.21	−0.08	0.26	0.11		
SMOKING	NS	NS	NS	NS	NS	NS	NS	NS	
Age	0.29	0.28	0.13	0.28	−0.41	−0.60	0.48	NS	NS

Correlation coefficients are significant at 0.05 levels (2-tailed).  NS: not significant.

**Table 3:** Association between eGFR_MDRD_ or eGFR_CG_ and adiponectin using linear regression analyses in non-diabetic West Africans.

Dependent variable (log eGFR_MDRD_)							Dependent variable (log eGFR_CG_)					
	Univariate model			Multivariate model			Univariate model			Multivariate model		
	*B*_MDRD_ (SE)	Beta_MDRD_	*P*_MDRD_	*B*_MDRD_ (SE)	Beta_MDRD_	*P*_MDRD_	*B*_CG_ (SE)	Beta_CG_	*P*_CG_	*B*_CG_ (SE)	Beta_CG_	*P*_CG_
log10AdipoQ	−0.09 (0.02)	−0.18	<0.0001	−0.05 (0.02)	−0.10	0.004	−0.15 (0.02)	−0.26	<0.0001	−0.05 (0.02)	−0.09	0.001
Age	—	—	—	−0.003 (0.00)	−0.26	<0.0001	—	—	—	−0.005 (0.0)	−0.47	<0.0001
Sex	—	—	—	0.01 (0.01)	0.04	0.26	—	—	—	0.04 (0.01)	0.14	<0.0001
BMI	—	—	—	0.00 (0.001)	−0.01	0.79	—	—	—	0.01 (0.001)	0.33	<0.0001
HTN	—	—	—	0.008 (0.01)	0.03	0.42	—	—	—	0.007 (0.01)	0.02	0.44
Smoking	—	—	—	0.01 (0.02)	0.02	0.52	—	—	—	0.01 (0.02)	0.02	0.41
log10LDL-C	—	—	—	−0.15 (0.03)	−0.17	<0.0001	—	—	—	−0.13 (0.03)	−0.14	<0.0001
log10HDL-C	—	—	—	−0.08 (0.02)	−0.13	<0.0001	—	—	—	−0.07 (0.02)	−0.10	<0.0001
log10TG	—	—	—	−0.21 (0.03)	−0.26	<0.0001	—	—	—	−0.17 (0.02)	−0.19	<0.0001

*B*_MDRD_: unstandardized coefficient for GFR estimated by MDRD method; *B*_CG_: unstandardized coefficient for GFR estimated by CG method.

Beta_MDRD_: standardized coefficient for GFR estimated by MDRD method; Beta_CG_: standardized coefficient for GFR estimated by MDRD method.

SE: standard error; AdipoQ: adiponectin.

HTN: hypertension; BMI: body mass index.

Adjusted *R*-square for univariate model = 0.03, adjusted *R*-square for multivariate model = 0.30 when eGFR_MDRD_ is the dependent variable.

Adjusted *R*-square for univariate model = 0.07; adjusted *R*-square for multivariate model = 0.56 when eGFR_CG_ is the dependent variable.

Significance level set at 0.05.

**Table 4:** Association between moderate CKD and adiponectin levels in non-diabetic West Africans using logistic regression models.

Models	Dependant variable: CKD (0, 1)^¥^										
	Predictor	CKD defined by eGFR_MDRD_					CKD defined by eGFR_CG_				
		*B*	S.E.	Wald chi-square	df	*P* value	*B*	S.E.	Wald chi-square	df	*P* value
Model 1: unadjusted	AdipoQ	1.48	0.50	8.95	1	0.003	1.84	0.38	22.9	1	<0.0001
Model 2: age and sex adjusted	AdipoQ	1.05	0.53	3.93	1	0.05	1.56	0.46	11.65	1	0.001
Model 3: age, sex, and serum lipids*	AdipoQ	1.61	0.59	7.52	1	0.006	1.90	0.48	15.38	1	<0.0001

^¥^CKD coding: 0, reference group represents subjects with eGFR ≥ 60 mL/min/1.73 m^2^; 1 represents subjects with eGFR < 60 mL/min/1.73 m^2^.

*Serum lipids in the model: HDL-C, LDL-C, triglycerides.

SE: standard error; B: logistic regression coefficient; df: degree of freedom; OR: odds ratio; AdipoQ: adiponectin.

Sex coding—0: male and 1: female.
